# Commentary on “Complications of Collagen Biostimulators in Brazil: Description of Products, Treatments, and Evolution of 55 Cases”

**DOI:** 10.1111/jocd.16751

**Published:** 2024-12-22

**Authors:** Fabiano Nadson Magacho‐Vieira

**Affiliations:** ^1^ Magacho Institute for Health Education Fortaleza Ceará Brazil; ^2^ Department of Clinical, Aesthetic and Surgical Dermatology Batista Memorial Hospital Fortaleza Ceará Brazil

**Keywords:** adverse effects, collagen stimulators, complications, fillers, poly‐l‐lactic acid

## Abstract

**Background:**

A recent study highlighted variability in complication rates among polylactic acid (PLA)‐based collagen stimulator fillers, with notably high rate of complications linked to PLLA‐Elleva. The study suggested that product‐specific characteristics might have greater impact on outcomes than injection techniques.

**Methods/Results:**

Through a critical analysis of pertinent literature, this commentary explores how PLLA‐Elleva's unique physicochemical properties, particularly its bimodal degradation process, may contribute to the increased nodule formation observed.

**Conclusion:**

Although PLLA‐Elleva's degradation pattern does not inherently compromise safety, it introduces variability that requires careful attention to dosage, injection technique, and patient selection.

Dear Editor,

I read “*Complications of Collagen Biostimulators in Brazil: Description of Products, Treatments, and Evolution of 55 Cases*” by Ianhez et al. [1], which examines complications associated with polylactic acid (PLA)‐based collagen stimulators in Brazil, with great interest. This study identifies nodules as the most common complication, occurring in 89.1% of cases, with 49.1% attributed to “PLLA‐Elleva,” also marketed internationally as Gana V (Gana R&D, Seongnam, Republic of Korea). The article highlights the variability in complications among different PLLA products and underscores the influence of product‐specific characteristics over injection techniques. In that context, I would like to draw attention to how the unique degradation process of this product may help explain those findings.

PLA biostimulators stimulate collagen synthesis through controlled hydrolytic degradation, releasing lactic acid and its oligomers [2]. The diffusion, degradation, and release of soluble oligomers, driven by matrix swelling, molecular properties (mass, conformation, chirality), and environmental factors (such as pH and temperature), are further influenced by differences in degradation kinetics shaped by polymer stereochemistry, molecular weight, crystallinity, and particle morphology, which can have a significant impact on clinical outcomes [2, 3]. While most PLA fillers exhibit uniform (bulk) degradation, PLLA‐Elleva exhibits a bimodal degradation pattern, with a second peak of particles with increased size dispersity emerging during the process [2, 4]. This heterogeneous degradation may create localized concentrations of degradation products, potentially contributing to a higher incidence of nodules [5]. This could provide a partial explanation for the higher nodule rates reported by Ianhez et al.

The article propositions underscore the importance of incorporating product‐specific considerations into clinical decision‐making. While PLLA‐Elleva's unique degradation pattern does not inherently compromise its safety or efficacy, it introduces an element of unpredictability that warrants careful attention to dosage, injection technique, and patient selection to optimize outcomes and mitigate risks.

Future studies should prioritize comparative analyses using advanced imaging techniques to monitor in vivo degradation and differentiate granulomas from product accumulations. Safety and efficacy could be further enhanced by developing product‐specific protocols tailored to their unique physicochemical profiles.

I hope this commentary encourages further dialogue and investigation into this critical aspect of cosmetic dermatology.

## Disclosure

Dr. F.N. Magacho‐Vieira serves as medical director for Derma Dream Corporation Brazil.

## Ethics Statement

No photos or patient personal information are in this manuscript. No institutional approval was required for this publication. The author confirms that this manuscript adheres to the ethical policies of the journal, as noted on the journal's author guidelines page.

## Conflicts of Interest

While this commentary focus solely on PLLA‐based products, Aesthefill, a poly‐D,L‐lactic collagen stimulator distributed in Brazil by Derma Dream Corporation, may be considered a competing product. The author has no current financial or professional affiliations with manufacturers or distributors of PLLA and did not receive any incentives or funding for this article.

## Data Availability

The author has nothing to report.
